# Single-Point Nail Sampling to Diagnose Onychomycosis Caused by Non-Dermatophyte Molds: Utility of Polymerase Chain Reaction (PCR) and Histopathology

**DOI:** 10.3390/jof9060671

**Published:** 2023-06-14

**Authors:** Aditya K. Gupta, Elizabeth A. Cooper, Tong Wang, Sara A. Lincoln, Wayne L. Bakotic

**Affiliations:** 1 Division of Dermatology, Department of Medicine, University of Toronto, Toronto, ON M5S 3H2, Canada; 2 Mediprobe Research Inc., London, ON N5X 2P1, Canada; lcooper@mediproberesearch.com (E.A.C.); twang@mediproberesearch.com (T.W.); 3 Bako Diagnostics, 6240 Shiloh Rd, Alpharetta, GA 30005, USA; slincoln@bakodx.com (S.A.L.); wbakotic@bakodx.com (W.L.B.)

**Keywords:** non-dermatophyte mold, dermatophyte, onychomycosis, diagnosis, polymerase chain reaction, PAS staining, molecular biology, histopathology

## Abstract

The three most commonly used methods for diagnosing non-dermatophyte mold (NDM) onychomycosis are culture, polymerase chain reaction (PCR), and histopathology. Toenail samples from 512 patients (1 sample/patient) with suspected onychomycosis were examined using all three diagnostic tests. A statistically significant association was found between PCR and histopathology results, as well as between fungal culture and histopathology results. All PCR-positive and culture-positive dermatophyte samples were confirmed by histopathology. However, 15/116 (12.9%) of culture-positive NDM samples had negative histopathology results, while all PCR-positive NDM samples were confirmed by histopathology. The overall rate of dermatophyte detection was higher using PCR compared to culture (38.9% vs. 11.7%); the lower rate of NDM detection by PCR (11.7% vs. 38.9%) could be attributed to the restriction of the assay design to seven pre-selected targets. When repeat sampling in the clinic is not possible, a combination of NDM detection by PCR and positive histopathology of hyphae may be a proxy for NDM infection, particularly where the NDM occurs without a concomitant dermatophyte. There was a high degree of correlation between negative PCR and negative histopathology. A negative PCR result with negative histopathology findings may be a reliable proxy for the diagnosis of non-fungal dystrophy.

## 1. Introduction

Onychomycosis is the most prevalent nail infection associated predominantly with dermatophyte fungi (*Trichophyton* spp., *Microsporum* spp., and *Epidermophyton* spp.) [1,2]. The diagnosis of onychomycosis is difficult, with microscopy and culture methods being the traditional techniques used for fungal detection [3]. These methods are readily available and relatively cheap for laboratories. However, both light microscopy (potassium hydroxide examination [KOH]) and culture show variable sensitivities for fungal detection. Culture in particular shows high rates of false negative detection and may require many weeks for fungal growth, which is required for genus/species identification [4]. For these reasons, KOH and culture have limited ability to provide an accurate and timely diagnosis for patients awaiting treatment.

Histopathology provides an in situ review of the nail plate and subungual keratin for fungal elements, providing direct evidence of fungal invasion that is not visible in a simpler light microscopic exam. Histopathology is associated with good accuracy in the diagnosis of onychomycosis but lacks the ability to provide genus-/species-level identification of the invading organism [4]. The utility of histopathology is therefore reduced for clinicians, as fungal organism identification is often preferred for the selection of the optimum treatment based on the etiologic agent detected. 

Polymerase chain reaction (PCR) and other molecular diagnostic methods are recognized as having a more rapid and sensitive identification than culture for the detection of dermatophytes, but adoption of these methods has not been widespread [5,6]. The cost of these methodologies is higher than culture, and the lack of automated, standardized techniques has held back commercial use of molecular diagnostics in this realm. Interpretation of PCR results is complicated by the problem of determining contaminant/commensal organisms from possible pathologic organisms and a lack of demonstrated viability of the detected organism. This is a particular problem for non-dermatophyte mold (NDM) species found in toenails, which are usually dismissed as environmental contaminants [7]. Traditionally, detection in repeated samples taken at sequential time points without growth of concomitant dermatophytes is the standard for diagnosing NDM nail infection by PCR or culture [8]. However, repeat sampling is usually impractical, rarely achieved, and can significantly delay patient treatment if pursued. 

In this study, we retrospectively assessed the data from 512 diagnostic samples collected from patients suspected of onychomycosis. Each sample was subjected to histopathology assessment, PCR testing, and fungal culture. We hypothesized that nail plate or subungual keratin showing fungal invasion by hyphae represented true disease, so a PCR-positive NDM in conjunction with histopathological evidence of hyphae presence could identify a significant pathogen. On the other hand, when the PCR was positive without a positive histopathology finding, this was more likely to be a contaminant, and repeat sampling would be advisable.

If these histopathology-positive species found by PCR also grew in culture, it can be presumed that the PCR finding represents a viable fungal species that may have a role in the continuing nail dystrophy.

The dataset presented in the study provides valuable information on how well histopathology correlates with PCR findings and how often positive PCR results may reflect viable fungi, and as such, may be a good proxy for determining when NDMs are ‘true’ agents of onychomycosis where repeat sampling cannot be obtained.

## 2. Materials and Methods

Between 4 August and 26 September 2022, nail samples were collected from patients with suspected onychomycosis attending podiatry and dermatology offices in the United States and sent to the laboratory for routine diagnosis (one sample per nail per person). Sample selection required sufficiently large portions of the nail to allow for all of the desired diagnostic procedures. Performed diagnostic procedures included a histopathological exam (using both a periodic acid–Schiff [PAS] reaction and a Grocott methenamine silver [GMS] stain examination when included in physician orders) and molecular diagnostic testing (PCR) for the identification of possible dermatophytes, saprophytes, yeasts, and bacteria. Standard mycologic culture was performed on the residual sample following case completion/reporting and subjected to de-identification of patient information. All samples were provided as part of a non-interventional standard-of-care diagnostic procedure by a qualified medical diagnostic laboratory and/or de-identified patient information, and as such, they do not represent a clinical trial for which an ethics overview and informed consent are required.

### 2.1. Histopathology Assessment

The collected nail samples were subjected to a histological examination via PAS or GMS staining when requested. All slides were reviewed by a dermatopathologist for diagnosis. Diagnostic information included the (a) presence or absence of fungal hyphae or yeast, (b) estimation of the quantity of fungal elements present, (c) pattern of nail involvement, and (d) additional pathologic findings. Visual assessment of the quantity of fungal growth within the examined tissue was categorized as follows: rare elements, minimal growth (<10%), moderate growth (10–80%), and florid growth (>80%). The pattern of infiltration of the nail unit keratin was also determined through direct visualization. Nail involvement was subdivided into subungual, superficial, and total dystrophic patterns. When the pattern of infiltration was unclear, an indeterminate designation was used. 

### 2.2. Culture

Samples were cultured on potato dextrose agar (PDA) and mycobiotic media, then incubated at 30 °C in ambient air for 2–4 weeks. Mass spectrometry (VITEK MS system) and lactophenol blue staining were used, as appropriate, to aid in confirming the microorganism present. The VITEK MS system utilizes matrix-assisted laser desorption/ionization time-of-flight (MALDI-TOF) technology. Protein extraction was performed in accordance with the protocol of a laboratory-developed test (LDT) approved by the College of American Pathologists and the New York State Department of Health (NYS DOH) (Project ID: 27083, PFI: 8490, approval date: 6 April 2012). Protein peaks were analyzed using the VITEK MS software, and organisms were identified using a U.S. Food and Drug Administration (FDA) approved database. 

### 2.3. Sample Homogenization and DNA Extraction

Specimens were transferred into bead tubes (Omni International, Kennesaw, GA, USA), followed by the addition of 1 mL of lysis buffer. Homogenization was performed first using a bead ruptor (Omni International, Kennesaw, GA, USA) for 5 minutes, followed by a 10 min incubation step at 90 °C in a dry bath, and a 2.5 min centrifugation step at 12,500 rpm [9,10]. Automated DNA extraction was carried out using the Mag-Bind Plant DNA DS Kit (Omega Biotek, Norcross, GA, USA) on a Hamilton Microlab STAR workstation. 

### 2.4. PCR Testing

DNA extracts from nail samples were tested using the BakoDx Onychodystrophy Infectious Agent Detection (OIAD) assay, a multiplex real-time PCR assay utilizing TaqMan technology for the detection of a specific genetic target. This assay was designed and validated in accordance with both Clinical Laboratory Improvement Amendments (CLIA) and NYS DOH standards. Specifically, primers and probes were subjected to in silico analysis and wet testing to ensure appropriate specificity; optimization of primers, probes, and thermocycling conditions was performed by testing combinations of various concentrations and annealing temperatures.

The OIAD assay consists of two sequential panels: detection and identification. The detection panel covers a broad range of targets in order to determine the presence, if any, of relevant dermatophytic and/or saprophytic fungi groups, yeasts, and bacteria (*Pseudomonas aeruginosa*). If a specimen produces a positive result for dermatophyte, saprophyte, and/or yeast, it is subsequently tested in a separate identification multiplex panel to help determine the species present. 

Targeted dermatophytic fungi species in the identification panel include the *Trichophyton mentagrophytes* complex (*T. interdigitale*, *T. tonsurans*, and *T. mentagrophytes*), the *T. rubrum* complex (*T. violaceum* and *T. rubrum*), *Microsporum canis*, *M. gypseum*, and *Epidermophyton floccosum*. Targeted NDM genera (saprophytic fungi) include *Acremonium*, *Alternaria*, *Aspergillus*, *Curvularia*, *Fusarium*, *Scopulariopsis*, and *Neoscytalidium*. Targeted yeast species include *Candida albicans*, *C. guilliermondii*, *C. parapsilosis* complex (*C. metapsilosis*, *C. orthopsilosis*, and *C. parapsilosis*), *C. tropicalis*, *Cryptococcus* spp., *Malassezia* spp., and *Trichosporon* spp.

### 2.5. Data Compilation and Analysis

Upon the completion of all diagnostic procedures, PCR testing and correlating fungal culture provided the identification of the following organism groups: dermatophytes, saprophytes, dermatophyte plus saprophytes, yeasts, and *Pseudomonas* (as the single bacterial agent reported). PCR and fungal culture findings were cross-referenced against histopathology outcomes of ‘Positive’ or ‘Negative’; ‘Positive’ histopathology was further subdivided into ‘Hyphae Positive’ or ‘Hyphae Negative’, where ‘Hyphae Negative’ indicated yeast or other non-hyphal histological findings. 

Data was tabulated using IBM SPSS Statistics package version 29.0.0.0 (241). Distributions of fungal group identifications versus histopathology outcomes were assessed using the Pearson Chi-square Test of Association, and Cramer’s V for degree of association when Chi-square was significant. 

## 3. Results

**Figure 1:**
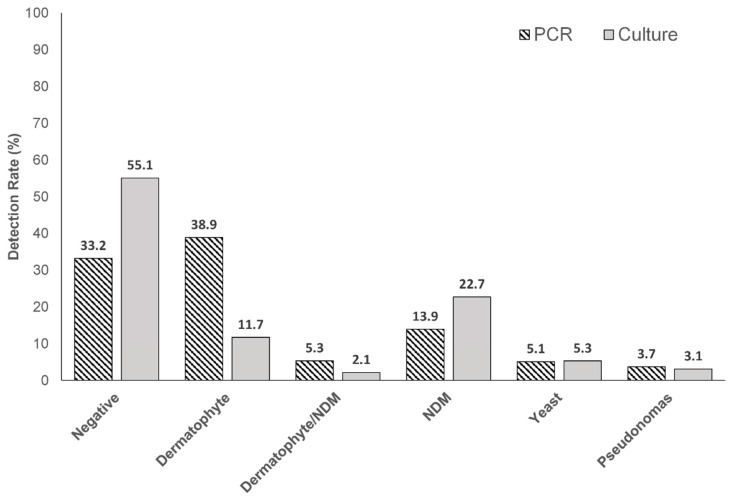
Detection rates of fungi (dermatophytes, NDMs, and yeast) in toenail samples suspected of onychomycosis by PCR and culture. NDM: non-dermatophyte mold; PCR: polymerase chain reaction.

Results of the PCR testing versus histopathology are shown in Table 1. Dermatophytes were found alone and in conjunction with saprophytes (NDM), in 38.9% (199/512) and 5.3% (27/512) of samples, respectively, by PCR. PCR detected dermatophytes at a three-fold higher rate than culture ([199/512] 38.9% versus 11.7% [60/512]) (Figure 1). 

**Figure 2:**
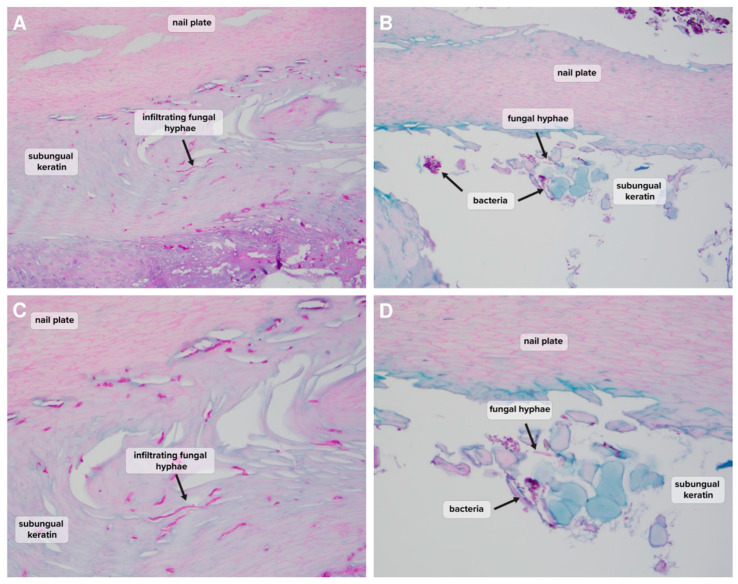
Histopathology assessment of NDM-positive mycotic toenail samples using PAS staining. (**A**,**C**) Moderate fungal growth detected with infiltrating hyphae in the subungual keratin ((**A**) PAS, original magnification ×20; (**C**) PAS, original magnification ×40). (**B**,**D**) In the case of onycholysis, rare fungal growth and bacterial colonization were observed in the subungual keratin ((**B**) PAS, original magnification ×20; (**D**) PAS, original magnification ×40).

**Figure 3:**
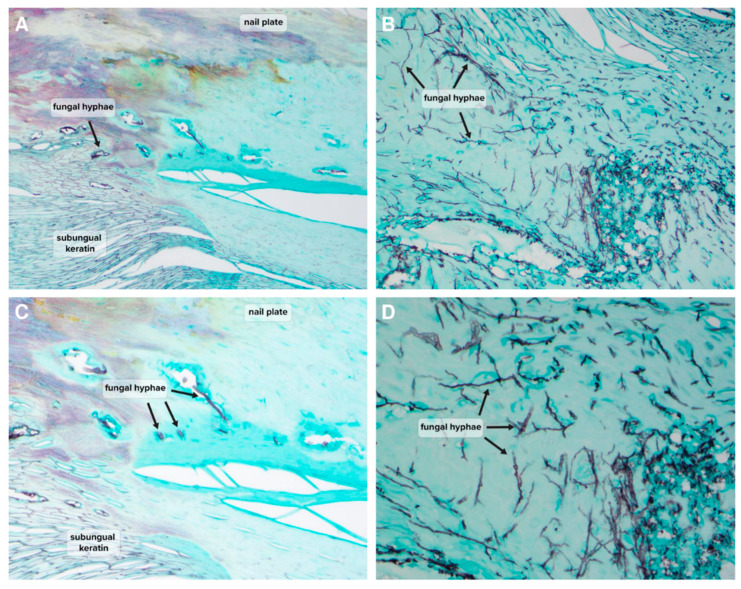
Histopathology assessment of NDM-positive mycotic toenail samples using GMS staining. (**A**,**C**) Minimal fungal growth detection ((**A**) GMS, original magnification ×20; (**C**) GMS, original magnification ×40). (**B**,**D**) Florid fungal growth detection ((**B**) GMS, original magnification ×20; (**D**) GMS, original magnification ×40).

Chi-square tests showed a significant association between PCR detection and histopathology detection, as well as culture detection and histopathology detection (Table 1 and Table 2). All PCR dermatophyte-positive and NDM-positive samples were histopathology positive, and furthermore, they were all indicated as hyphae-positive (Table 1). Examples of histopathological findings demonstrating the presence of hyphae in NDM-positive samples are shown in Figure 2 and Figure 3. The high rate of association between PCR and histopathology-positive detection gives strength to the hypothesis that detection of dermatophytes or NDMs by PCR is reflective of the true fungal nail presence of the detected species. Interestingly, where PCR results were negative, 85.9% (146/170) of samples were also histopathology-negative, and no significant fungal species were found for any histopathology-negative samples (17 *Pseudomonas* detections considered likely nail contaminants) (Table 1). Nail dystrophy may lead to secondary infections and associated discoloration. In contrast to positive PCR findings suggesting true nail invasion, cases of negative histopathology with negative PCR may provide a good proxy for likely non-fungal nail dystrophy. 

Culture detection also showed a statistically significant association with histopathology results. All positive dermatophyte cultures were histopathology positive for hyphae (60/60), but the low overall rate of positive detection of dermatophytes by culture (11.7% [60/512] versus 38.9% by PCR [199/512]) limits the utility of culture to identify dermatophytes over PCR (Table 1 and Table 2). The negative outcome associations are much lower for culture versus PCR as well, with only 48.6% (137/282) of negative cultures showing negative histopathology (Table 2). The reduced value of culture detection compared to PCR likely reflects the increased risk of contaminant detection, decreased sensitivity, different growth requirements for dermatophytes compared to NDMs/yeasts, and possible culture overgrowth of NDMs/yeasts, which prevents efficient detection of dermatophytes. 

Both PCR and culture detection results showed a relatively low prevalence of yeasts (5.1% [26/512] and 5.3% [27/512]) and *Pseudomonas* (3.7% [19/512] and 3.1% [16/512]) (Table 1 and Table 2). The detection of hyphae in these samples by histopathology can likely be attributed to sampling artifacts, since only a portion of the submitted patient sample is subjected to each of the three diagnostic tests. Additionally, the restriction of PCR testing to pre-selected targets, especially in the case of NDMs where seven genera are incorporated into the assay design, may have led to the misidentification of specimens. Although *Candida* spp. has exhibited the ability to grow hyphae, the morphological transition from the yeast form to the hyphal form requires specific microenvironmental conditions such as hypoxemia and the presence of serum [11]. Given that these conditions are unlikely to be found on the epidermis or the nail plate, further studies are warranted to elucidate their pathogenic potential in onychomycosis.

Considering NDM detection by PCR and culture without concomitant dermatophytes, culture had a higher rate of detection (22.7% [116/512] versus PCR (13.9% [71/512]), which likely reflects that species detectable by culture had no limit, whereas PCR was limited to the pre-selected seven NDM genera in the validated PCR protocols (*Acremonium*, *Alternaria*, *Aspergillus*, *Curvularia*, *Fusarium*, *Scopulariopsis*, and *Neoscytalidium*) (Table 1 and Table 2). As for the possibility of environmental contaminants, we were particularly interested in the finding that 100% [71/71] of the NDMs detected by PCR without an associated dermatophyte were also found to be histologically positive (Table 1). Of the 98 NDM-positive PCR findings (NDM alone [N = 71] or with a dermatophyte [N = 27]), 60 samples were also culture-positive for NDMs (61.2% [60/98], Table 3), but only 45 culture samples (45.9% [45/98]) showed the same NDM genus in both PCR and culture. These 45 cultures confirm the viability of NDMs detected by PCR and provide some confidence that PCR-identified organisms with positive histopathology often represent ‘clinically significant’ fungi. As the PCR detection range of NDMs is restricted to seven genera, the ability of PCR to provide agreement with culture is also restricted, and this agreement rate is likely an underestimate of the utility of PCR for NDM detection. 

‘Clinically significant’ also does not necessarily indicate ‘causative’, but the positive infiltration of nail keratin observed by histopathology would seem to indicate that NDMs are a live presence within the nail keratin, which may be contributing to ongoing nail dystrophy (Figure 2). Of the 45 agreements, 35 of them (77.8%) occur as solo NDM PCR organism detections, and these include the strains previously reported as potential pathogens in onychomycosis: *Fusarium*, *Scopulariopsis*, and *Neoscytalidium*. (Table 4) [12,13,14]. Where agreement occurs in samples with combination dermatophyte/NDM detection, the species are those most frequently considered environmental contaminants that may be present secondary to the primary dermatophyte infection (*Alternaria* and *Curvularia*). 

The identification of PCR- or culture-positive dermatophyte isolates is presented in Table 5. All dermatophytes detected by PCR (N = 199) and culture (N = 60) were also positive for hyphae by histopathology (Table 1 and Table 2), further confirming their clinical significance in onychomycosis. Consistent with previous studies, *T. rubrum* appears to be the most prevalent dermatophyte species in North America, followed by *T. mentagrophytes* [15,16]. Two cases of possible mixed infections were detected by PCR, whereas none were detected by culture. Competitive outgrowth of one infecting organism over another may contribute to the lack of sensitivity of the culture method [15]. Four samples were not speciated, likely due to the etiological agent not being one of the pre-selected targets in the identification panel (i.e., *T. mentagrophytes* complex, *T. rubrum* complex, *M. canis*, *M. gypseum*, and *E. floccosum*).

## 4. Discussion

Traditionally, any dermatophyte detection, whether through PCR or culture, and regardless of microscopy, has been considered the ‘causative pathogen’ for dermatophytosis cases [17]. Per our data, we found dermatophyte detections by PCR were highly associated with the detection of fungal hyphae in histopathology examination, providing strong evidence that a single PCR detection of a dermatophyte is indeed detecting a pathogenic organism (Table 1). Culture-positive detection also correlated with positive histopathology, but overall detection rates were much lower than PCR, reducing the value of this correlation for cultures (Table 2).

Our data corroborates other findings that PCR provides higher rates of detection of dermatophytes versus culture [9,18]. Though rates of NDM detection appear lower with PCR, this is likely due to the restrictions of PCR testing to seven NDM genera. NDMs here are underestimated; per other studies, a much wider variety of species is likely detectable in toenails [19,20,21,22]. Broader molecular detection of NDM species in correlation with histopathology would provide a better determination of the utility of single NDM detection for predicting the positive presence of NDMs in nail samples. 

The limitations of culture detection prevent widespread investigation of the full spectrum of organisms in both normal and infected toenails [23]. Instead, a wide variety of molecular tools are now providing information on the nail mycobiome and the relationship of fungi to other organisms in healthy and diseased states. There is evidence to suggest that fungi exist in conjunction with bacteria as a ‘balanced’ commensal microbiome in many areas of the body, with bacteria aiding the fungi in avoiding host immunity and pharmaceutical treatment through biofilms [18,19,23,24,25]. This study had limited NDM and bacterial detection and therefore cannot support any suggestion of bacterial co-existence with fungi. However, based on medical literature, we may expect to detect a wider variety of fungi and bacterial species in nails than has been reported by culture methods. Wider investigation by molecular methods is necessary to improve the understanding of organismal presence/interactions in onychomycosis pathology and response to treatment [19].

To determine a significant NDM nail infection, repeat sampling has been deemed necessary, but obtaining multiple samples can be difficult and significantly delay diagnosis. Single PCR NDM detections have been criticized for being possible contaminations rather than causative agents. In this study, we proposed that a positive PCR result for NDMs found in conjunction with positive histopathology without any dermatophyte detection represents a ‘clinically significant’ organism in the nail, and we found that all PCR-positive NDM species in this data met the ‘clinically significant’ criteria (Table 1). It is important to note that these two methods are complementary and not mutually exclusive, as histopathology alone cannot provide genus- or species-level identification, while PCR detection alone cannot indicate the viability of the organism [3]. When repeat sampling is not possible, a combination of PCR NDM detection and positive histopathological detection of hyphae may be a suitable proxy for possible NDM infection, particularly where the NDM occurs without a concomitant dermatophyte.

Though the focus of detection is on finding possible infectious organisms, this data shows a high correlation between negative PCR and negative histopathology. It is well known that only 50% of toenail dystrophies have been linked to a fungal infection [26]. The low reliability of culture diagnosis complicates the determination of fungal versus non-fungal dystrophy. Our data suggests that a negative PCR may be a reliable proxy for the diagnosis of non-fungal dystrophy when histopathology is also negative. These findings complement other diagnostic data, which noted that negative PCR was a better proxy for onychomycosis cure after topical treatment than negative culture [5].

In the midst of an ongoing antifungal resistance epidemic associated with superficial fungal infections, it has become increasingly important to perform a timely and accurate diagnosis prior to the initiation of treatment [10,27]. A recent survey conducted in the United States found that only 15.3% of physicians perform confirmatory lab testing for patients suspected of onychomycosis; the rate was higher for dermatologists (31%), compared to podiatrists (16.9%) and general practitioners (5.2%), with the most common testing method being histopathology (12%), followed by culture (2.8%) and PCR (2.1%) [28]. Concurrently, the antifungal resistance epidemic has reached countries outside of its initial outbreak location, including Canada, Greece, and Japan [27]. A recent study had reported two initial cases inside the U.S. (December 2021 to March 2023); treatment failure had occurred in both cases following use of oral terbinafine, and one case was suspected to be the result of local transmissions [29]. In the same period, we detected mutations conferring decreased terbinafine susceptibility in the toenail samples of U.S. onychomycosis patients [10]. As terbinafine is currently the most commonly prescribed antifungal agent due to its low cost, the spread of resistance development to this agent has the potential to incur significant healthcare burdens [28]. Faced with this impending challenge, healthcare professionals may benefit by adopting the practice of antifungal stewardship and incorporating routine confirmatory testing into their practice [30]. With the exception of patients presenting with pre-existing mechanical damages to the nail matrix, appropriate antifungal treatment will increase the likelihood of clinical improvements and patient satisfaction, in addition to minimizing unnecessary antifungal exposure leading to the selection of resistance mutations [31].

Limitations to the use of molecular diagnostics include the use of pre-selected detection targets, contamination, a lack of viability indication, as well as general availability and cost [3]. The utility of molecular techniques in onychomycosis is supported by the demonstrated superiority of the technology over traditional fungal culture [9,18]. Several elements may dictate the cost profile of a given test, including utilization profiles, automation, and, as is the current case with onychomycosis, conscientious assay design [32]. As with any newer technology, increased adoption over time tends to lead to reduced production costs and improved cost effectiveness of specific components. While PCR technologies are relatively well automated currently, multiplex assay designs and continual assay improvements act in conjunction to lower the overall cost burden of this technology while improving diagnostic capabilities. The current assay design includes fungal organisms most likely to be pathogenic but does not include a complete coverage of organisms found in the nail mycobiome, as the latter would be cost prohibitive. With the increasingly recognized role of other potential pathogenic organisms in onychomycosis and the need for expansion of detection targets to optimize treatment, newer technologies can be employed by physicians to maintain an acceptable cost profile with improved diagnostic accuracy.

Another limitation of the current data set is the possible bias associated with sample selection. Analyzed samples were based on the physician’s ordering patterns, which included histochemical examination and PCR evaluation. The larger size of toenail samples required for completion of all diagnostic procedures (PCR, culture, and histopathology) could potentially overrepresent patients with higher disease severity, although the degree of any impact on the conclusions drawn is uncertain. The aim of this study was to interpret the diagnostic testing methods through both direct comparisons and cross-referencing with histopathology; therefore, we feel that any potential bias would have minimal impact and represent a form of ever-present sampling bias.

## 5. Conclusions 

Despite its higher cost, PCR diagnosis of onychomycosis has demonstrated improved utility over fungal culture, particularly when used in conjunction with histopathological evaluation of the nail plate and subungual keratin. The current data demonstrates that when repeat sampling is not possible, a combination of PCR NDM detection and positive histopathological detection of hyphae may be a suitable proxy for possible NDM infection, particularly where the NDM occurs without a concomitant dermatophyte. A negative PCR may be a reliable proxy for the diagnosis of non-fungal dystrophy when histopathology is also negative. More access to molecular methods is needed to widen clinicians’ ability to diagnose onychomycosis, both prior to and following treatment. Improved diagnosis is also essential for expanding our knowledge of the nail mycobiome and our clinical understanding of onychomycosis pathology.

## Figures and Tables

**Table 1:** Detection rates of organism groups by PCR and histopathology.

PCR	Histopathology ^1,2^			Row Total
	Any Positive	Positive for Hyphae	Negative	
**Negative (N = 170)**	24/170 (14.1%)	24/24 (100%)	146/170 (85.9%)	170/512 (33.2%)
**Dermatophyte (N = 199)**	199/199 (100%)	199/199 (100%)	0	199/512 (38.9%)
**NDM (N = 71)**	71/71 (100%)	71/71 (100%)	0	71/512 (13.9%)
**Dermatophyte + NDM (N = 27)**	27/27 (100%)	27/27 (100%)	0	27/512 (5.3%)
**Yeast (N = 26)**	26/26 (100%)	9/26 (34.6%)	0	26/512 (5.1%)
***Pseudomonas* (N = 19)**	2/19 (10.5%)	2/2 (100%)	17/19 (89.5%)	19/512 (3.7%)
Total	349/512 (68.2%)	332/512 (64.8%)	163/512 (31.8%)	Total N = 512

^1^ Significant positive association of histopathology with PCR group detection: Chi-square test of association 408.772, df = 5, Sig *p* < 0.001; Cramer’s V 0.894 *p* < 0.001. ^2^ Significant positive association of hyphae with PCR group detection: Chi-square test of association 87.921, df = 5, Sig *p* < 0.001; Cramer’s V 0.870, *p* < 0.001. NDM = non-dermatophyte mold; PCR = polymerase chain reaction.

**Table 2:** Detection rates of organism groups by culture and histopathology.

Culture	Histopathology ^1,2^			Row Total
	Any Positive	Positive for Hyphae	Negative	
**Negative (N = 282)**	145/282 (51.4%)	138/145 (95.2%)	137/282 (48.6%)	282/512 (55.1%)
**Dermatophyte (N = 60)**	60/60 (100%)	60/60 (100%)	0	60/512 (11.7%) ^3^
**NDM (N = 116)**	101/116 (87.1%)	99/101 (98.0%)	15/116 (12.9%)	116/512 (22.7%)
**Dermatophyte + NDM (N = 11)**	11/11 (100%)	11/11 (100%)	0	11/512 (2.1%) ^3^
**Yeast (N = 27)**	22/27 (81.5%)	14/22 (63.6%)	5/27 (18.5%)	27/512 (5.3%)
***Pseudomonas* (N = 16)**	10/16 (62.5%)	10/10 (100%)	6/16 (37.5%)	16/512 (3.1%)
Total	349/512 (68.2%)	332/512 (64.8%)	163/512 (31.8%)	Total N = 512

^1^ Significant association of histopathology with culture group detection: Chi-square test of association 91.148, df = 5, *p* < 0.001; Cramer’s V 0.422 *p* < 0.001. ^2^ Significant association of hyphae with culture group detection: Chi-square test of association 93.221, df = 5, Sig *p* < 0.001; Cramer’s V 0.427, *p* < 0.001. ^3^ All dermatophyte cultures were also PCR-positive for dermatophytes. NDM = non-dermatophyte mold.

**Table 3:** Non-dermatophyte mold detection by PCR versus culture.

Culture	PCR		Row Total
	Positive (N = 98) ^1^	Negative (N = 414)	
**Positive (N = 127)**	60/98 (61.2%) ^2,3^ 45/98 (45.9%) ^2,4^	67/414 (16.2%)	127/512 (24.8%)
**Negative (N = 385)**	38/98 (38.8%)	347/414 (83.8%)	385/512 (75.2%)
**Total**	98	414	Total N = 512

^1^ Includes NDM-positive and mixed NDM/dermatophyte positive samples. ^2^ 100% of positive samples were also positive for histopathology. ^3^ Matching NDM organism-level detection per culture and PCR results. ^4^ Matching NDM genus-level detection per culture and PCR results. NDM = non-dermatophyte mold.

**Table 4:** Identification of NDMs in histopathology-positive samples confirmed by PCR and culture results (N = 45 *).

NDM Genus	Total	Mixed Detection	
		NDM Only	NDM + Dermatophyte
*Acremonium*	0/45 (0%)	-	-
*Alternaria*	6/45 (13.3%)	2/6 (33.3%)	4/6 (66.7%)
*Aspergillus*	17/45 (37.8%)	16/17 (94.1%)	1/17 (5.9%)
*Curvularia*	11/45 (24.4%)	6/11 (54.5%)	5/11 (45.5%)
*Fusarium*	9/45 (20%)	9/9 (100%)	-
*Scopulariopsis*	2/45 (4.4%)	2/2 (100%)	-
*Neoscytalidium*	3/45 (6.7%)	3/3 (100%)	-

* Of the histopathology-positive samples, NDM detection was positive in 60 samples by PCR and culture; of these 60 samples, detection of the same NDM genus was observed in 45 samples (48 total genus-level agreements).

**Table 5:** Identification of dermatophyte isolates detected by PCR or culture and confirmed by histopathology.

Dermatophytes	PCR (N = 199)	Culture (N = 60)
*T. rubrum* complex	172/199 (86.4%)	45/60 (75%)
*T. mentagrophytes* complex	18/199 (9.0%)	10/60 (16.7%)
*Microsporum*	1/199 (0.5%)	-
*Epidermophyton*	2/199 (1.0%)	1/60 (1.7%)
Mixed detection		
*T. rubrum/T. mentagrophytes*	1/199 (0.5%)	-
*T. mentagrophytes*/*Epidermophyton*	1/199 (0.5%)	-
Unidentified	4/199 (2.0%)	4/60 (6.7%)

## Data Availability

Supporting data are available from the corresponding author upon reasonable request.
